# Ectopic Eruption of Maxillary First Permanent Molars: Preliminary Results of Prevalence and Dentoskeletal Characteristics in Spanish Paediatric Population

**DOI:** 10.3390/children8060479

**Published:** 2021-06-06

**Authors:** Alexandra Helm, Andrea Martín-Vacas, Pedro Molinero-Mourelle, Antonia M. Caleya, Nuria E. Gallardo, María Rosa Mourelle-Martínez

**Affiliations:** 1 Department of Dental Clinical Specialties, Faculty of Dentistry, Complutense University of Madrid, 28040 Madrid, Spain; alexhelm@ucm.es (A.H.); amcaleya@ucm.es (A.M.C.); negallar@ucm.es (N.E.G.); mrmourel@ucm.es (M.R.M.-M.); 2 Eastman Dental Institute, University College London, London 28040, UK; 3 University Dental Clinic, “Alfonso X El Sabio” University, 28037 Madrid, Spain; 4 Department of Conservative Dentistry and Orofacial Prosthodontics, Faculty of Dentistry, Complutense University of Madrid, 28040 Madrid, Spain; pedromol@ucm.es; 5 Department of Reconstructive Dentistry and Gerodontology, School of Dental Medicine, University of Bern, 3010 Bern, Switzerland

**Keywords:** tooth eruption, ectopy, eruption, molar, first permanent molar, orthodontics interceptive, patient care planning, children

## Abstract

The ectopic eruption of the maxillary first permanent molar (EEM) is a local alteration of dental eruption with a multifactorial aetiology. The aims of our study were to determine the prevalence of the EEM in children and to analyse whether there is a relationship between EEM and dento-skeletal characteristics. A total of 322 children were analysed with the Ricketts cephalometric study and descriptive and analytical statistical analysis was carried out. The prevalence of EEM was 8.7%, with no statistically significant differences regarding gender or location, but a higher prevalence in the 7-year-old age group (18.8%) and bilateral EEM was more prevalent than unilateral EEM (*p* < 0.05). The most frequent findings were a shortened anterior cranial base, a retroposition of the maxilla and a distal position of the upper permanent first molar in relation to the pterygoid vertical in children with EEM. No statistically significant differences were found regarding the cephalometric parameters except a decreased palatal plane in the bilateral EEM group and a distal upper incisor position in the EEM group (*p* < 0.05). In conclusion, the prevalence of the EEM was 8.7%, more frequently bilateral, and significantly in seven-year-old patients. Children with bilateral EEM have decreased palatal plane values and a more posterior position of the upper incisor.

## 1. Introduction

The ectopic eruption of the maxillary first permanent molar (EEM) is defined as a local alteration of eruption, usually involving a mesial eruptive trajectory of the maxillary permanent first molar, which is blocked under the distal area of the temporary second molar. As a consequence, root resorption of the second primary molar occurs [1], even leading to its premature loss, with the consequent loss of space in the dental arch [2]. The EEM can be reversible or irreversible and the self-correction rate is highly variable, ranging from 6.25% to 91% [1,3].

Nowadays, EEM is understood as a pathological disorder of multifactorial aetiology, although its causes are not fully elucidated. The prevalence of EEM ranges from 0.75 to 11.8%, with a prevalence up to four times higher in patients with cleft lip and palate [4]. It can be considered a genetic or hereditary component due to a higher prevalence described among siblings (19.8%), and a recessive hereditary pattern has even been suggested in girls [5,6]. While some authors cite a higher incidence in boys, [1,5] others find no statistically significant differences between genders [7,8,9]. At the beginning of the 20th century, Chapman stated that, for EEM to occur, the mesial migration of the first permanent molar must be greater than the movement towards occlusal, suggesting the following aetiological factors: lack of mesial migration of the primary teeth and the bone that houses them, premature mesial migration of the first permanent molar, or premature eruption of the first permanent molar [10]. In addition, predisposing factors such as a larger than average tooth size of the maxillary primary or permanent teeth, larger affected permanent first and/or temporary second molar, a smaller maxilla, a posterior position of the maxilla in relation to the cranial base, late calcification of the affected permanent first molar, abnormal eruption angulation of the permanent first molar or poor bone growth in the region of the maxillary tuberosity have been proposed [9,11,12].

Early diagnosis of EEM can be made in children aged between five and seven years of age on routine radiographic examination, showing the first permanent molar more apical and mesial. During the eruption process, there are clear signs of resorption of the roots of the primary second molar. The first clinically observable sign is the inclination of the distal occlusal plane at the level of the primary second molar, which can lead to an anterior open bite [4,13]. In addition, there is a delay in the eruption of the permanent first molar and as it erupts, the distal cusps are usually observed first [3,14].

Due to the multifactorial aetiology of EEM, some authors have analysed the dento-skeletal characteristics associated with this disorder, however, there are few studies that have determined the association between skeletal malocclusion and EEM based on cephalometric analysis. Most of these studies conclude that although the aetiology of EEM is multifactorial, there are factors on which EEM depends, including maxillary hypoplasia, a more posterior position of the maxilla in relation to the skull base and an anomalous angle of eruption of the upper first permanent molar [9,10,11,12,15,16].

The aims of our study were to determine the prevalence of the ectopic eruption of the maxillary first permanent molar in the paediatric population and to analyse whether there is a relationship between this anomaly and dento-skeletal characteristics using Ricketts cephalometry.

## 2. Materials and Methods

### 2.1. Study Design and Ethical Aspects

A cross-sectional radiographic study was approved by the Ethics Committee of the Hospital Clínico San Carlos following the Declaration of Helsinki’s ethical principles for medical research involving human subjects. Informed consent from patients and/or their legal guardians was obtained from all participants before conducting the research.

### 2.2. Study Population

The sample was obtained from patients attending an oral and maxillofacial radiology centre in Madrid, Spain by non-probabilistic random sampling, and consisted of 322 children aged 6 to 9 years in first-stage mixed dentition, with digital orthopantomography and lateral teleradiograph of the skull and Ricketts cephalometric study [17]. The sample size calculation was carried out with the mathematical formula (population size: 100, confidence level: 95%, error range: 5%). At the collaborating radiology centre, approximately 100 radiographs of the age range used in our study were performed in three months, which provided a sample size of 81 patients in each age group. Finally, after eliminating the missing patients, each age group consisted of 80 patients. Exclusion criteria were as follows: patients with any systemic pathology, genetic syndrome or congenital orofacial malformation and/or patients with atypical resorption of the temporary second molar due to a cause unrelated to the eruption of the upper first permanent molar. The following study groups were established: Group 1: 6-year-old boys (*n* = 40), Group 2: 6-year-old girls (*n* = 40), Group 3: 7-year-old boys (*n* = 40), Group 4: 7-year-old girls (*n* = 40), Group 5: 8-year-old boys (*n* = 40), Group 6: 8-year-old girls (*n* = 40), Group 7: 9-year-old boys (*n* = 42) and Group 8: 9-year-old girls (*n* = 40).

### 2.3. Radiological Analysis

All radiographs were taken under the same technical specifications, and examined by the same calibrated operator. The software used for the cephalometric studies was Gioconda Ortoceph©. The radiographs were analysed with a 13” monitor, the zoom was set at 100%, and in case of doubt, the image was enlarged to 110%. A maximum of 30 radiographs were analysed per session and all the radiographs were taken under the same technical specifications (Table 1).

Atypical or pathological resorption was considered when a radiolucent “punch” image was found in the distal corono-radicular area of the second primary molar, due to the eruption of the first permanent molar, regardless of whether the latter had emerged or not.

The parameters of interest were then obtained from the Ricketts cephalometric study for each child: facial convexity, lower facial height, first molar position, upper incisor position, upper incisor inclination, facial depth, facial axis, maxillary depth, maxillary height, palatal plane, cranial deflection and anterior cranial length.

### 2.4. Statistical Analysis

The statistical analysis was performed by using the software IBM-SPSS-22 (IBM Corp. Released 2013. IBM SPSS Statistics v 22.0 for Windows; Armonk, NY, USA). A descriptive and analytical analysis of the variables obtained was carried out. Pearson’s chi-square test (χ^2^) was used to analyse the differences between the MES and the study subgroups (gender, age and cephalometric parameters). The analysis of variance test (ANOVA) with Duncan’s test as a post-hoc test was carried out to study the differences between inter- and intra-group quantitative measures. A 95% confidence interval was used, and a statistical significance level of 5% was used for all tests (*p* < 0.05). A randomly selected 20% of each group was measured in order to analyse intra-examiner reliability.

## 3. Results

A total of 322 patients who met the inclusion and exclusion criteria were analysed. Of the included patients 162 were boys (50.3%) and 160 girls (49.7%). Regarding age distribution, 80 patients were included in the 6-year, 7-year and 8-year groups (24.8%) and 82 patients in the 9-year group (25.5%).

### 3.1. Ectopic Eruption and Gender

EEM occurred in 28 of the 332 patients, giving a prevalence of 8.7%. Of the patients with EEM, 16% were boys and 12% girls, with no statistically significant differences (*p* > 0.05) (Table 2).

### 3.2. Ectopic Eruption and Age Group

Regarding the age group distribution, the highest prevalence was reported in the 7-year-old group, with 18.8% and statistically significant differences (*p* = 0.000) with respect to the no EEM group (control group). In decreasing order of prevalence, this was followed by the group aged 6 years (11.3%), 8 years (2.5%) and 9 years (2.4%), all with no statistically significant differences (*p* > 0.05) (Table 3).

### 3.3. Ectopic Eruption Location

The prevalence of bilateral EEM was 5.6% (18 patients) of the total sample, and unilateral EEM 3.1% (10 patients). Of the cases with EEM, 64.3% were bilateral and 35.7% unilateral, with no statistically significant differences between gender and in location of the EEM (*p* > 0.05). With respect to age groups, a statistically significant relationship was only found between the 7-year age group and other age groups and between the prevalence of bilateral EEM (13.8%) and unilateral (5%) EEM (*p* = 0.001), with no significant differences in the other groups studied.

### 3.4. Ectopic Eruption and Cephalometric Parameters

The most relevant cephalometric parameter results are summarized in Table 4. No statistically significant differences were found between the EEM group and the control group (*p* > 0.05), or between the mean intergroup and intragroup values of uni- and bilateral EEM (*p* > 0.05) in the groups studied, with the exception of the palatal plane values and the position of the upper incisor, that present altered values, explained below.

- The facial convexity values were slightly increased in the EEM group.
- There was a slight increase in lower facial height in the EEM group.
- The upper first molar was on average 0.3 mm more posterior with respect to the vertical pterygoid in children without EEM.
- The upper incisor had a position 0.7 mm more anterior in the group of children without EEM, with significant differences with respect to patients with EEM (*p* < 0.05), and there was a variability of almost 1 mm regarding its inclination.
- Facial depth tended to be negative in patients with EEM, while in patients without this pathology it was positive, with a mean difference greater than 1 mm.
- The facial axis was 1 mm increased in the EEM group.
- The maxillary depth is increased compared to the control group.
- Maxillary height presented a higher mean value in children without EEM.
- The palatal plane presented similar values between patients with and without EEM (*p* < 0.05). Intergroup differences were found (*p* = 0.017), determining that the difference was significant in the bilateral EEM group with respect to the unilateral EEM and control groups, finding lower values in the bilateral EEM in comparison to the other groups studied.
- Cranial deflection had a higher mean value in the group with EEM.
- The anterior cranial length was higher in the group without EEM, with a difference greater than 1 mm.

## 4. Discussion

EEM can be diagnosed early by appropriate clinical and radiographic examination. Direct comparison between studies was not possible as the research parameters (age, method of radiographic analysis, cephalometric measurements etc.) were variable. Comparing the present results with those previously reported, prevalence (Table 5) shows a large variability (0.75–11.8%), due to different patient inclusion criteria, sample size, diagnostic methods and assessment criteria [18].

Regarding age, while in other studies the sample ages ranged from 4 to 12 years, we set the age range of the sample at 6 to 9 years. The age range was chosen on the basis of the mean age at eruption of the first permanent molar and the age at the end of the eruptive stage of mixed dentition first phase. Analysing the laterality of this pathology, Sweet [23] found that EEM occurs more frequently bilaterally, coinciding with our results, where we observed that bilateral ectopic eruption is more frequent than unilateral eruption. Nevertheless, other authors, such as O’Meara et al. [22] have reported contrary findings. Respecting the relationship between EEM and dento-skeletal characteristics, it has been described in the literature that the inclination of the upper permanent first molar is an important factor in the aetiology of EEM [9,10,24], although other authors such as Nikiforuk have disagreed with this theory [25]. Bjerklin et al. analysed the position of the maxillary permanent first molar with lateral skull teleradiographs, as in our study, although they did not use Ricketts’ cephalometry but the reference lines of Björk and Solow, finding that the first permanent molar has an increased mesial angle and greater rotation in cases with EEM [22,26,27]. Pulver et al. also reported that the first permanent molar presents an atypical angle when its eruption is ectopic [9,12].

Canut and Raga used Ricketts cephalometry, as in our study, observing that the molar in the group with EEM is more distal in relation to the pterygoid vertical [16]. The present findings are in agreement with the previous studies mentioned, although without statistical significance, since in both unilateral and bilateral cases of EEM, the first molar was in a more posterior position compared to the control group.

Several authors have suggested that short jaw length may play an important role in EEM. Pulver describes that children with EEM have a smaller maxilla compared to the control group, suggesting that the decreased size of the maxilla and its more posterior position in relation to the skull base may be involved in the aetiology of EEM [9]. Bjerklin, in agreement with Pulver, found that there is a tendency for the length of the maxilla to be decreased [12]. Rah et al., in agreement with previous authors, concluded that poor jaw growth has an impact on EEM [16,20].

Mucedero et al. analysed the skeletal characteristics of the jaws of children with EEM using digitized models, also finding that the jaw length is shortened in the group of children with EEM, in agreement with previous studies [19]. On the other hand, Chintakanon and Boonpinon, as well as other authors, determined the maxillary length, without finding significant differences between the EEM group and the group without this pathology [7].

In addition to a short maxillary length, EEM could be related to a retrognathic maxilla. Canut and Raga found significant differences in the value of facial convexity between children with and without EEM, determining the presence of maxillary retrognathism in patients with EEM [16]. In the present study, similar mean values of facial convexity were found between children without and with EEM, with a slight increase in the value in the case of the presence of EEM, being statistically non-significant, in agreement with Chintakanon and Boonpinon [7]. Canut and Raga also found a tendency towards dolichocephaly, together with a posterior rotation of the chin, seeming to point to the existence of a shortened anterior cranial base in the case of children with EEM [16]. This finding is consistent with what it was found in the present study, with the smallest values for anterior cranial length being found in the group of children with EEM, especially in the case of children with unilateral EEM, who had the smallest values, although without statistical significance.

Regarding to the palatal plane, Canut and Raga found increased values in the case of EEM, although the data were not statistically significant [16]. The mean value of the palatal plane among children with EEM in our study was higher than in the group without ectopic eruption, and this finding was statistically significant, particularly in children with bilateral EEM.

The results of this study indicate that there could be dento-skeletal differences in patients with EEM in comparison with those without EEM. Although in early-diagnosed patients, a preventive orthodontic or orthopedic treatment should be arranged, in order to prevent more severe pathology. Due to the limitation of the low prevalence of this disease, our sample of patients with EEM was small, especially in the eight- and nine-year-old group, and for this reason, statistical tests of cephalometric values were not carried out differentiating age groups. The results of both this and previous studies should be interpreted as an indicator, or predisponent factor for EEM. It would be necessary to be able to increase the sample in order to evaluate the differences in cephalometric values and age groups. It could be interesting to arrange a multicenter study, in order to increase the study sample, and to study racial variations in the prevalence of the EEM, and its relationship with dento-skeletal anomalies.

## 5. Conclusions

The ectopic eruption of the maxillary first permanent molar prevalence is 8.7%, being higher in boys (16%) than in girls (12%), more frequently bilateral than unilateral, and significantly higher in seven-year-old patients. The most frequent findings were a shortened anterior cranial base, a retroposition of the maxilla, and a more distal position of the upper permanent first molar in relation to the pterygoid vertical in children with ectopic eruption. Children with bilateral ectopic eruption have decreased palatal plane values and a more posterior position of the upper incisor.

## Figures and Tables

**Table 1:** Radiograph technical specifications.

Orthopantomography ^1^	Lateral Teleradiography ^2^
Nominal voltage: 208/220/230/240 V	Nominal voltage: 380 V
Rated current: 12 A	Tube: Comet, rotating anode
Frequency: 50/60 Hz	Tube voltage: 50–125 kV
Tube stream: 9–16 mA	Tube stream: 25–300 mA
Aluminum equivalent filter 2.5 mm	Focus-midline distance: 152 cm
Focal size 0.5 × 0.5 mm	Focal size: 0.3 × 0.3 mm
Medium technique used: 65 kV y 12 mA	Medium technique used: 80 kV y 20 mA
Fixed time: 12 s	Midline-image receptor distance: 12 cm

^1^ Siemens Orthopantomograph, Ortofox^®^; ^2^ SEDECAL.

**Table 2:** Ectopic eruption of the maxillary first permanent molar. Gender distribution.

			EEM		Total
			No	Yes	
**Gender**	**Male**	*N* (%)	146 (90.1)	16 (9.9)	162 (100)
	**Female**	*N* (%)	148 (92.5)	12 (7.5)	160 (100)
**Total**		*N* (%)	294 (91.3)	28 (8.7)	322 (100)

**Table 3:** Ectopic eruption of the maxillary first permanent molar. Age distribution.

			EEM		Total
			No	Yes	
**Age group (years)**	**6**	*N* (%)	71(88.8%)	9 (11.3%)	80 (100%)
	**7**	*N* (%)	65 (81.3%)	15 (18.8%) *	80 (100%)
	**8**	*N* (%)	78 (97.5%)	2 (2.5%)	80 (100%)
	**9**	*N* (%)	80 (97.6%)	2 (2.4%)	82 (100%)
**Total**		*N* (%)	294 (91.3%)	28 (8.7%)	322 (100%)

* *p* chi-square of Pearson test ≤ 0.05.

**Table 4:** Cephalometric parameter mean values and ectopic eruption of the maxillary first permanent molar.

	Non EEM	Unilateral EEM	Bilateral EEM	Chi-Square (sig ^+^)	ANOVA (sig)
Facial convexity	1.00 ± 2.52	1.20 ± 2.71	1.27 ± 2.38	0.357	0.886
Inferior facial height	−3.15 ± 4.32	−2.70 ± 2.42	−3.55 ± 4.37	0.363	0.875
Upper molar to TPV	1.66 ± 2.95	0.79 ± 1.84	0.76 ± 2.12	0.281	0.304
Upper incisor position	2.10 ± 3.08 *	1.02 ± 1.97	0.95 ± 1.92	0.046 *	0.167
Upper incisor angulation	0.81 ± 9.05	1.74 ± 6.47	−0.01 ± 5.96	0.145	0.876
Facial depth	0.59 ± 3.36	−0.52 ± 1.98	−0.92 ± 3.51	0.494	0.114
Facial axis	−1.14 ± 4.35	−2.42 ± 3.76	−1.75 ± 4.82	0.343	0.569
Maxillar depth	−0.01 ± 3.87	−0.03 ± 3.05	−0.36 ± 2.74	0.660	0.926
Maxillar height	4.30 ± 3.46	3.97 ± 3.72	3.51 ± 3.88	0.525	0.622
Palatal plane	1.77 ± 3.15	2.28 ± 1.76	−0.32 ± 2.14 *	0.145	0.017 *
Cranial deflection	1.18 ± 2.09	1.86 ± 2.26	0.94 ± 2.15	0.494	0.526
Anterior cranial length	1.19 ± 3.14	−0.05 ± 3.00	0.14 ± 3.02	0.406	0.193

* Statistical significance for the chi-square of Pearson and ANOVA test *p* < 0.05. ^+^ Sig. Statistical test significance

**Table 5:** Ectopic eruption of the maxillary first permanent molar prevalence data [1,5,7,8,9,11,16,19,20,21,22].

Author, Year	Sample Size	Cases (%)
Cheyne, 1947	500	9 (1.8)
Young, 1957	1619	52 (3.2)
O’Meara, 1961	315	6 (2.0)
Pulver, 1968	831	26 (3.1)
Bjerklin, 1981	2903	126 (4.3)
Kimmel, 1982	5277	202 (3.8)
Canut, 1983	800	26 (3.3)
Chintakanon, 1998	3612	27 (0.8)
Barberia-Leache, 2005	509	22 (4.3)
Mucedero, 2005	1052	26 (2.5)
Rah, 2017	786	93 (11.8)
Present study	322	28 (8.7%)
